# A Case Report of Adrenocortical Adenoma Mimicking Congenital Adrenal Hyperplasia in a Young Girl

**DOI:** 10.1097/MD.0000000000001046

**Published:** 2015-06-26

**Authors:** Qingfeng Sheng, Zhibao Lv, Weijue Xu, Jiangbin Liu, Yibo Wu, Zhengjun Xi

**Affiliations:** From the Department of General Surgery (QS, ZL, WX, JL, YW); Department of Pathology (ZX), Children's Hospital of Shanghai, Shanghai Jiao Tong University. No. 24, Lane 1400, West Beijing Rd, Shanghai, PR China

## Abstract

Adrenal cortical tumors are rare in children. Secondary tumors associated with untreated congenital adrenal hyperplasia (CAH) have also been reported in pediatric population. It is difficult for pediatricians to differentiate these 2 lesions.

We described a 4.5-year-old girl who presented with symptoms and signs of virilization. Bone age was 9.5 years. Genetic analysis of *CYP21A2* and *CYP11B1* revealed a heterozygous mutation of *CYP11B1* at c.1157C>T (A386V). No germline *p53* gene mutation including R337H was detected.

The patient was first misdiagnosed as CAH and treated with hydrocortisone for 3 months. Diagnosis of an adrenal cortical tumor was confirmed by laboratory data and abdominal computed tomography. After resection of the tumor, serum steroids normalized and clinical signs receded. The child received no additional treatment and remains disease free after 12 months of close observation. Histological examination showed neoplasia cells with predominantly eosinophilic cytoplasm and few atypical mitotic figures. The proliferation-associated Ki-67 index was <1% detected by immunohistochemistry.

Neoplasm is a rare but significant cause of precocious puberty (PP). The possibility of neoplasms should always be considered early to avoid delayed cancer diagnosis and treatment in cases of PP.

## INTRODUCTION

The mean prevalence of adrenal incidentalomas from the literature is 2.3% (range, 1%–8.7%) at autopsy and 0.64% (range, 0.35%–1.9%) at computed tomography (CT) scan.^1^ However, adrenocortical tumors (ACTs) are rare in children, comprising <0.2% of all childhood neoplasms. The most frequent clinical presentation is virilization, alone or in combination with hypercortisolism.^2,3^ Congenital adrenal hyperplasia (CAH) may also present with peripheral precocious puberty (PP). And secondary tumors associated with untreated or inadequately treated CAH have been reported in both adult and pediatric population.^4,5^ Therefore, it is not easy for pediatricians to differentiate the above 2 conditions. Herein, we reported a 4.5-year-old girl with a virilizing adrenal cortical adenoma whose steroid studies and genetic analysis supporting the diagnosis of CAH, but after complete removal of the tumor, the hormonal abnormalities normalized and clinical signs receded.

## CASE REPORT

**FIGURE 1:**
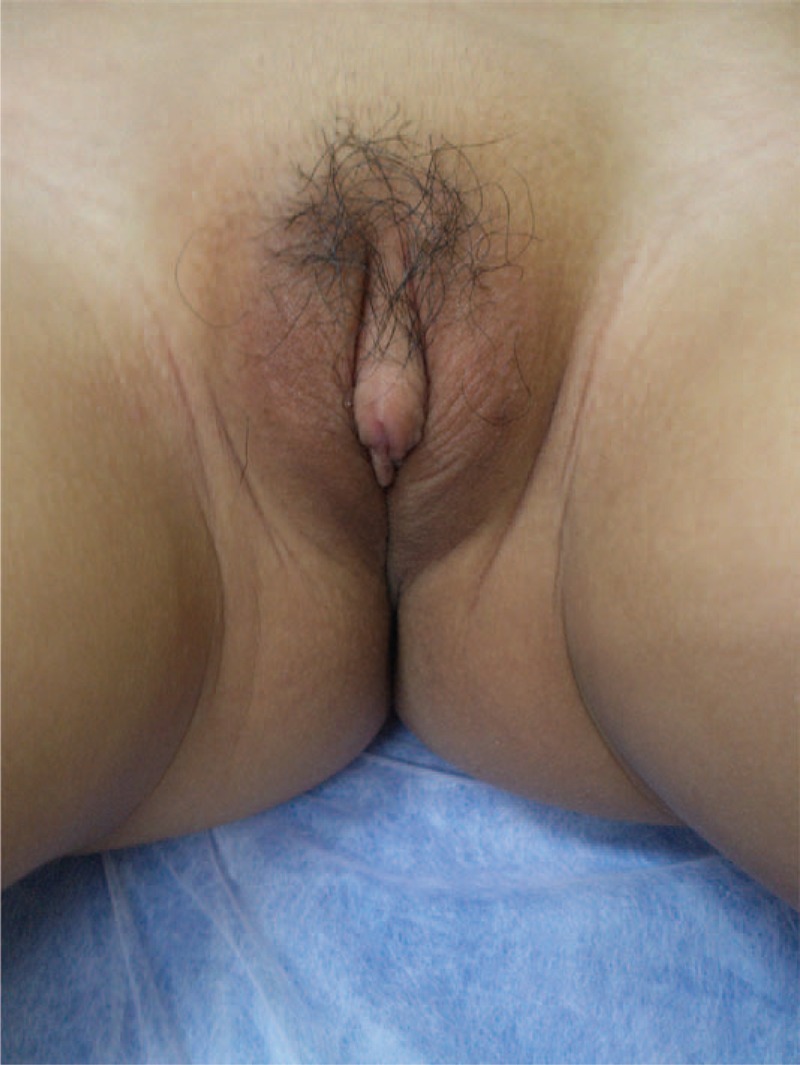
Clinical photography of the 4.5-year-old girl showing pubic hair and clitoromegaly.

**FIGURE 2:**
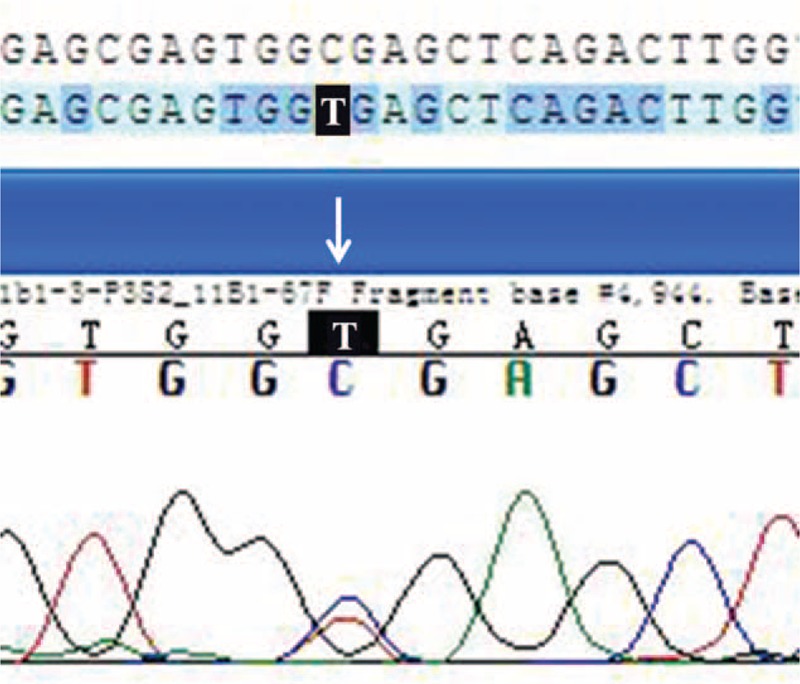
Direct sequencing of *CYP11B1* showing the heterozygous A386V mutation (arrow, nucleotide substitution GCG-GTG).

Ethical approval was obtained from the Ethics Board of the Children's Hospital of Shanghai, Shanghai Jiao Tong University. Written informed consent was obtained from the patient's parents on behalf of the child. A 4.5-year-old girl was referred with a 10-month history of pubic hair and a deep voice. Retrospectively, accelerated growth rate was diagnosed (growth velocity 12 cm/y). The girl's height was 114.5 cm (97th percentile) with a weight of 24.5 kg (>99th percentile). On physical examination (Figure 1), she was found to have pubic hair, clitoral hypertrophy (measuring 1.8 cm), deepening of the voice. There was no acne, no axillary hair, no breast development, no vaginal discharge, and no labial fusion. Bone age was 9.5 years. Blood pressure was 90/55 mm Hg. Genomic DNA was extracted from peripheral blood leukocytes using a commercial kit (Blood & Cell Culture DNA Mini Kit, Catalog No.: 13323, QIAGEN GmbH, Germany). Mutation analysis of *CYP21A2* and *CYP11B1* by sequencing the polymerase chain reaction amplification products of exons revealed a heterozygous mutation of *CYP11B1* at c.1157C>T (A386V, the alanine at position 386 was substituted by a valine, Figure 2). Procedures (DNA isolation, amplification, purification, and sequencing) were performed according to the instructions described in the manufacturer's protocol. Initial serum steroid measurement (Table 1) and genetic study suggested the diagnosis of CAH. The girl was treated with hydrocortisone (20 mg/d) for 3 months. Because the elevated level of 17-hydroxy-progestrone, testosterone, and dehydroepiandrosterone was not suppressed, the treatment was discontinued.

**TABLE 1:**
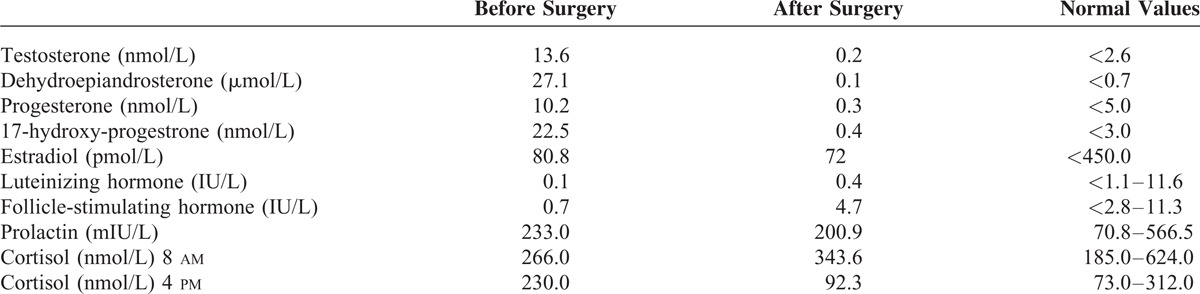
Serum Steroid Profiles Before and After Surgery

**FIGURE 3:**
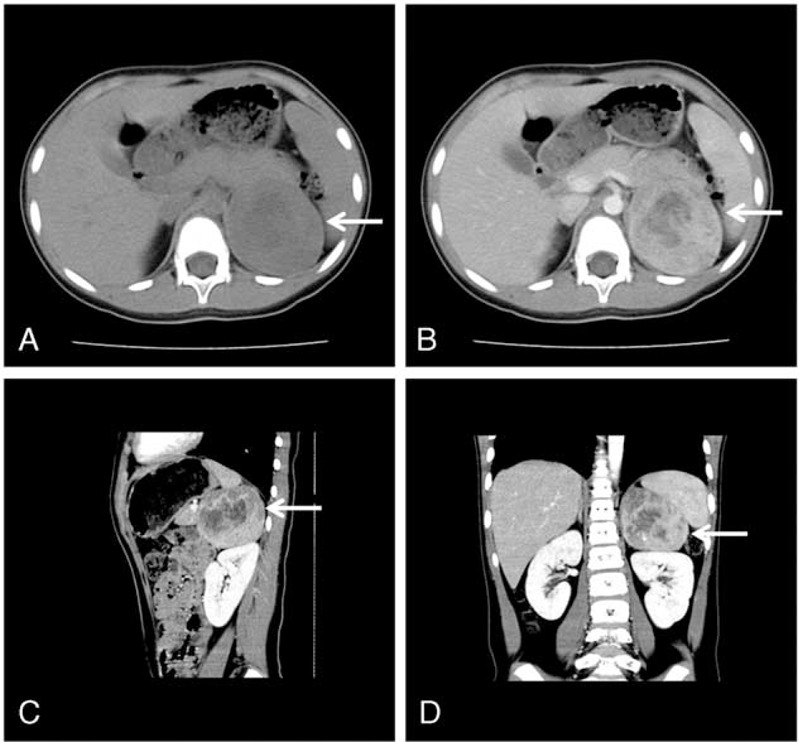
CT scan showing an ovoid tumor (arrow) with mixed signal intensity, calcification, and inhomogenous enhancement. Axial view (A) and intravenous contrast-enhanced axial (B), sagittal (C), and coronal (D) views. CT = computed tomography.

**FIGURE 4:**
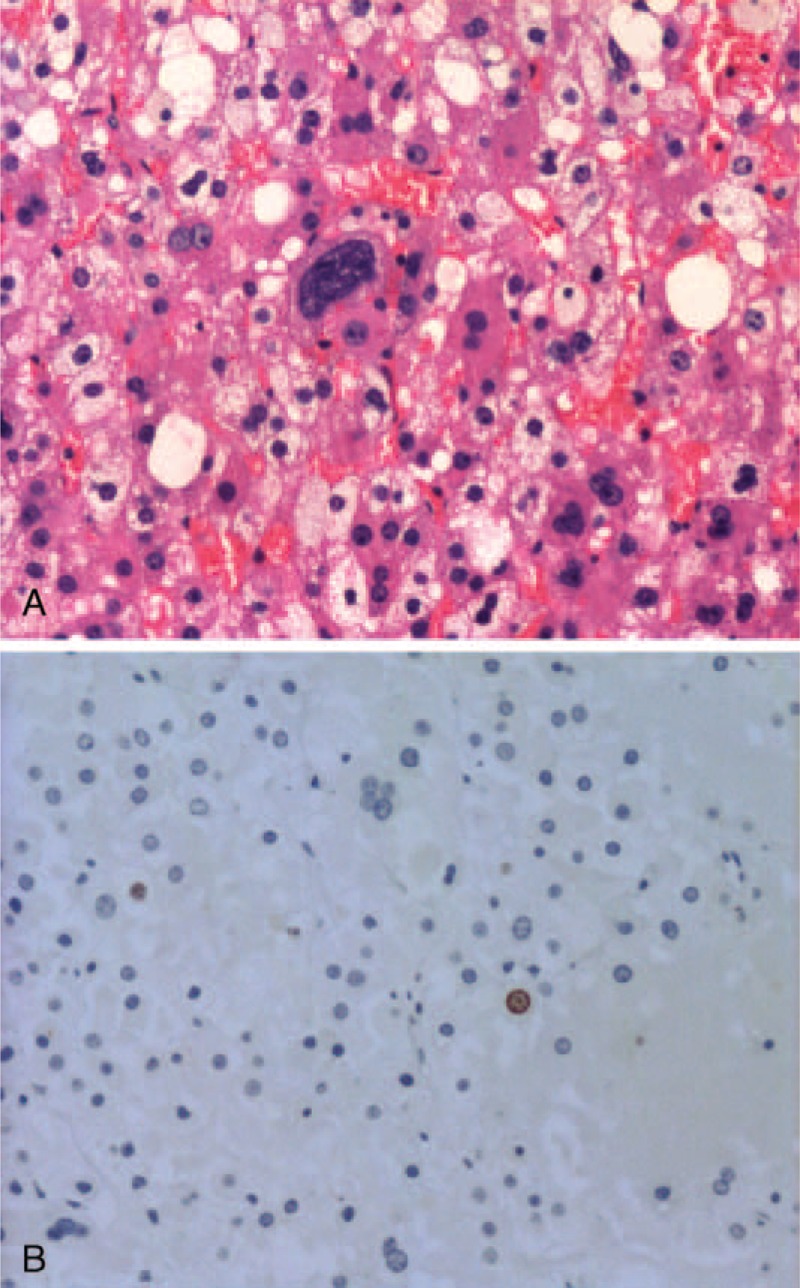
(A) Histopathological examination demonstrating adrenal cortical tumor cells with abundant eosinophilic cytoplasm and slightly irregular nuclei (H&E, original magnification × 200). (B) Immunohistochemical staining showing extremely low level of Ki-67 expression (original magnification ×200). H&E = hematoxylin and eosin.

The diagnosis was reevaluated in September 2013. Abdominal CT scan showed an adrenal tumor on the left side with a size of 6.2 cm (Figure 3). The well-defined tumor has a regular margin, calcification, mixed signal intensity, and inhomogenous enhancement after intravenous contrast. No distant metastasis (lung, liver) was noted. A left adrenalectomy was performed and an encaspsulated mass weighing 95 g (6.5 × 6.0 × 7.0 cm) was completely removed. Histological examination revealed neoplasia cells with predominantly eosinophilic cytoplasm, slightly irregular small size nuclei (nuclear grade 2), and few atypical mitotic figures (Figure 4). No tumor necrosis, vascular or capsular invasion was found. Adrenal cortical adenoma was diagnosed according to the criteria of Weiss et al.^6^ The proliferation-associated Ki-67 index (the percentage of Ki-67 positive cells) was <1% detected by immunohistochemistry (Figure 4). The procedures of immunostaining were performed as we previously described.^7^ No germline *p53* gene mutation including R337H (chromosomal locus 17p13) was observed using DNA sequencing. The postoperative course was uneventful. After surgery (1 month) the serum steroids normalized and clinical signs receded. There was no evidence of recurrence within a follow-up of 12 months.

## DISCUSSION

The underlying cause of peripheral PP (independent of gonadotropin and maturation of the hypothalamic–pituitary–gonadal axis) in girls is usually benign or unclear. Kaplowitz^8^ reported that the 2 most common conditions were premature adrenarche and premature thelarche. Assessment of PP is complex, and cancer is often underestimated. Primary organ lesions included brain tumor (pineal or optic pathway/hypothalamic tumor), ACT (adenoma or carcinoma), and hepatoblastoma, ovarian tumor (granulosa-theca cell tumor, Sertoli-stromal cell tumor, teratoma, etc.).^9,10^ Another serious cause of PP in early childhood is late presentation of CAH (21-hydroxylase or 11β-hydroxylase deficiency). A small group of patients with ACTs might be misdiagnosed as CAH because the size of tumor was small in early stage and serum hormonal studies were misleading. It is crucial to differentiate whether the adrenal cortical tumor is primary or secondary to untreated CAH as a consequence of adrenocorticotropic hormone over-secretion. The latter always shows satisfactory regression after steroid therapy and needs no surgery. Therefore, it may be necessary to repeat abdominal imaging study when ACTs are suspected especially in developing countries where children often receive appropriate medical treatment late in the course of disease.

Unfortunately, histopathological distinction between benign and malignant lesions remains difficult. The Weiss scoring system might be of some value in predicting malignant behavior.^6,11^ The Ki-67 index can help differentiate adenomas from carcinomas. Although high frequency of *p53* mutation (especially R337H) has been reported in southern Brazil,^3,12^ this is not the case in our study. Radical removal of the complete tumor by open or laparoscopic surgery is the mainstay of treatment. No adjuvant therapy was administrated due to the unavailability of mitotane in China. The overall prognosis of adrenal adenoma is excellent, in contrast to adrenal cortical carcinoma (especially stages III and IV).^3,11,13,14^ There is always a delay between the onset of symptoms and accurate diagnosis in children with PP. So, the possibility of neoplasm should be considered early to avoid delayed cancer diagnosis and treatment.

In summary, we described an adrenal cortical adenoma mimicking CAH in a young girl. Neoplasm is a rare but significant cause of PP. ACTs might be misinterpreted as CAH. Although the girl in the present study seems to have been cured, long-term follow-up is warranted.
